# Carbon Black Functionalized with Serinol Pyrrole to Replace Silica in Elastomeric Composites

**DOI:** 10.3390/polym16091214

**Published:** 2024-04-26

**Authors:** Federica Magaletti, Martina Galbusera, Davide Gentile, Ulrich Giese, Vincenzina Barbera, Maurizio Galimberti

**Affiliations:** 1 Department of Chemistry, Materials and Chemical Engineering “G. Natta”, Politecnico di Milano, Via Mancinelli 7, 20131 Milan, Italy; federica.magaletti@polimi.it (F.M.); martina.galbusera@mail.polimi.it (M.G.); davide.gentile@polimi.it (D.G.); 2 Deutsches Institut für Kautschuktechnologie e. V., Eupener Straße 33, 30519 Hannover, Germany; ulrich.giese@dikautschuk.de

**Keywords:** pyrrole methodology, functionalization, elastomer composites, Payne effect, hysteresis

## Abstract

Elastomer composites for dynamic mechanical applications with a low dissipation of energy are of great importance in view of their application in tire compounds. In this work, furnace carbon black functionalized with 2-2,5-dimethyl-1*H*-pyrrol-1-yl-1,3-propanediol (SP) was used in place of silica in an elastomer composite based on poly(styrene-co-butadiene) from solution anionic polymerization and poly(1,4-cis-isoprene) from *Hevea Brasiliensis*. The traditional coupling agent used for silica was also used for the CB/SP adduct: 3,3′-bis(triethoxysilylpropyl)tetrasulfide (TESPT). The composite with the CB/SP + TESPT system revealed a lower Payne effect, higher dynamic rigidity, and lower hysteresis, compared to the composite with CB + TESPT, although the latter composite had a higher crosslinking density. The properties of the silica and the CB/SP + TESPT-based composites appear similar, though in the presence of slightly higher hysteresis and lower ultimate properties for the CB/SP-based composite. The use of CB in place of silica allows us to prepare lighter compounds and paves the way for the preparation of tire compounds with lower environmental impacts.

## 1. Introduction

Tires are of fundamental importance for the mobility of people and goods, and they can play a key role in the development of sustainable transport, which is a pillar of climate action, which is the Sustainable Development Goal n. 13 in the 2030 Agenda for Sustainable Development [1]. The annual global freight and passenger traffic volumes were forecasted by the United Nations to grow by 50% and 70%, respectively, from 2015 to 2030. It is estimated that the cars on the road will be about 2.4 billion [2] and the tires will be 3.4 billion units [3]. Tires contribute around 20 percent of a vehicle’s carbon footprint, and about 90% of this contribution comes from the use stage of a tire [4]. The carbon emission of a car tire during its use ranges from 550 kg CO_2_ eq to 840 kg CO_2_ eq [5] and is mainly due to the rolling resistance (RR), which signifies “the energy consumed per unit distance of travel as a tire rolls under load” [6].

**Figure 1:**
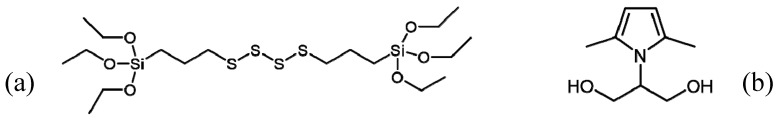
Chemical structures of bis(triethoxysilylpropyl)tetrasulfide (TESPT) (**a**) and 2-(2,5-dimethyl-1*H*-pyrrol-1-yl)-1,3-propanediol (serinol pyrrole, SP) (**b**).

Reinforcing fillers [7,8,9] are responsible for most of the hysteresis of a rubber compound for tires; hence, of the rolling resistance of a tire, the filler networking causes the dissipation of energy. Precipitated silica [10,11] is the preferred filler for a rubber compound with low hysteresis [12,13,14]. Indeed, silica is a nanostructured filler, with a high surface area and activity and with silanols on its surface, which allow a reaction with molecules able to favor compatibilization with the hydrocarbon matrix and, most of all, the reaction with elastomer chains. The covalent bond between the elastomer and silica, established via a coupling agent, reduces filler networking [7,8,9], hence the hysteresis of a compound. A sulfur-based silane, such as bis(triethoxysilylpropyl)tetrasulfide (TESPT), is the coupling agent most widely used [15,16], also at the industrial scale. Its chemical structure is shown in Figure 1.

Silica, however, presents important drawbacks, such as a high compound viscosity, which leads to worse processability and a shorter shelf life, and the need for higher energy and special equipment for mixing. Moreover, silica is corrosive and abrasive, and its adduct with silane is adhesive with the metal surfaces of mixers. Furthermore, the condensation of silanols with TESPT releases ethanol, which is then burnt to carbon dioxide. It has been estimated [17] that 8.44 × 10^4^ tons of CO_2_ are released worldwide per year because of the use of TESPT in a tire tread and that the emission value of volatile organic compounds (VOCs) due to the use of coupling agents in the tire industry is 130,000 m^3^ [18]. Research for the reduction in VOC emissions is thus essential for the sustainable development of the rubber industry [19,20,21]. Finally, silica-based compounds have no electrical conductivity, and this is a major drawback for an application such as the one in tire compounds.

The best way to avoid the problems of silica would be to use carbon black [22], which is a nanostructured, high-surface-area reinforcing filler, easily compatible with the elastomer matrix, with lower supramolecular interactions compared to silica and which has been widely used in the rubber world since the beginning of the last century. Moreover, CB has a lower density than silica; hence, its use at the same volume fraction leads to lighter rubber compounds. However, CB cannot establish a chemical bond with elastomer chains, and this leads to remarkable compound hysteresis. The solution lies in the functionalization of CB. However, it is acknowledged [23,24] that the functionalization reactions of CB are troublesome and are often based on dangerous chemicals and harsh reaction conditions. Some of the authors reported the pyrrole methodology [24,25,26,27,28] as a simple and versatile method, characterized by a high atom efficiency and a very low amount of waste, to covalently graft a variety of functional groups on a sp^2^ carbon allotrope. In brief, a domino reaction occurs [27]: a pyrrole compound (PyC), obtained from the neat reaction of a primary amine with 2,5-hexanedione, undergoes carbocatalyzed oxidation in the benzylic position and the oxidized PyC gives rise to a cycloaddition reaction with the carbon substrate. Adducts of CB were prepared with pyrrole compounds containing sulfur-based functional groups [24], reactive with unsaturated elastomer chains, such as thiol -SH and disulfide -SS- groups. It was shown that CB adducts with sulphurated PyC, used in a hybrid filler system with CB in an elastomer composite based on unsaturated elastomers, led to more efficient crosslinking [29], with an increase in dynamic rigidity of about 20% and to a reduction in hysteresis of about 10% at 70 °C, with similar/better ultimate tensile properties. However, it was not reported if these adducts could replace silica. Adducts of CB were prepared as well with 2-(2,5-dimethyl-1*H*-pyrrol-1-yl)-1,3-propanediol (serinol pyrrole, SP) as PyC [26]. Its chemical structure is shown in Figure 1. The CB/SP adduct was used for the partial replacement of silica in a composite based on unsaturated elastomers, and TESPT was used as the coupling agent. More efficient crosslinking, a lower Payne effect, and lower hysteresis were obtained for the composites with CB/SP. The OH groups appeared to facilitate the interaction with silica and with silane, and hence with the polymer chains. Head space analysis suggested the occurrence of the reaction of silane with CB/SP. However, a complete substitution of silica was not performed. Hence, the objective of substituting silica with a CB/PyC adduct in an elastomeric composite suitable as a tire compound, obtaining the same dynamic–mechanical properties, has not yet been achieved.

The objective of this work was to use a CB/SP adduct as the reinforcing filler of an elastomer composite suitable for a tire-tread compound, at least with the aim of reproducing the properties of a silica-based composite, without using silica. The CB/SP adduct was prepared according to the reported procedure [26] and was characterized by means of thermogravimetric analysis, nitrogen adsorption measurements with the Brunauer, Emmet, and Teller (BET) technique, and with scanning electron microscopy (SEM). Poly(1,4-*cis*-isoprene) from *Hevea Brasiliensis* (Natural Rubber, NR) and poly(styrene-co-butadiene) from anionic solution polymerization were the elastomers used. The CB/SP-based composite was compared with composites containing either pristine silica or pristine CB by using the same volume fraction of the fillers and the same processing procedure. The CB grade was CBN234, and the silica was a precipitated silica with a high surface area (165 m^2^/g). TESPT was used in all the composites, assuming it could act as a coupling agent for the silica and CB/SP. The aim of this study was to verify the ability of CB/SP to behave as silica, and the issue of ethanol release was neglected. The crosslinking density and the curing kinetics, the dynamic–mechanical properties in the shear, and in the axial mode and tensile properties were investigated. To evaluate the interaction between the CB and CB/SP fillers with the elastomer chains, the Kraus equation was used by applying the equilibrium swelling method [30,31].

The advantages of using CB in place of silica have been reported above. However, it could be argued that CB is not the ideal filler for the sustainable development of elastomer composites, as it is a material from a fossil fuel. Fortunately, other families of carbon materials, either biobased or circular, are appearing on the scene and could become useful reinforcing materials for elastomeric composites. The most important are the chars from the pyrolysis of end-of-life (EOL) tires [32] or from a biomass (biochar) [33]. It is worth analyzing if these chars can already fulfill the requirements of high-performance elastomer composites. In the scientific literature, rubber composites with either char from EOL tires [34,35,36,37,38] or from biochar [39,40,41,42] were reported. The former carbon materials were compared with furnace CB, such as CBN330 [35], N550 [36,37], and N660 [33], also by using combinations of them [34], in elastomers such as EPDM [35] and SBR [34,35,37]. A smaller size [36], a similar [35,36,37] or larger [34] surface area, and a lower structure [35,37] were observed. It has been said [38] that char from EOL tires is a significant challenge because of its impurities. A reduction in the tensile modulus at 300% [37] was reported. The tensile strength [34] and the tear strength [35,37] were higher for the furnace CB, and the detachment of rubber chains during breakage and lower reinforcement were observed in the presence of the char from tires [36]. In conclusion, it was stated that the char from tires can be used for rubber formulations [37], but CB was found to be a more active filler [34,35]. Biochar was obtained from different sources, such as lignin [41], wood-derived waste biomass [40], and agricultural waste from citrus trees trim [42], and was used in composites based on NR [40] and SBR [41,42], in comparison with CBN330 [40,41]. Biochar is said to have a higher ash content, lower carbon content, larger particle size, and more surface functional groups, [39,40], though the grade from lignin [41] could have a high specific surface area with a mesoporous structure. The elasticity, the strengthening impact at higher elongations, and the tensile strength of rubber samples filled with biochar were found to be lower [40]. Only 25% of CB could be replaced with biochar without losing its mechanical properties [43]. In a nutshell, the following comment was reported for biochar, which also appears to be valid for char from EOL tires: the successful use “relies on the ability to modify its properties” [39]. The pyrrole methodology could indeed modify the properties of these more sustainable carbon materials, making them suitable as fillers for elastomer composites. In light of this perspective, our research aimed at demonstrating the potential of the pyrrole methodology, working with traditional furnace carbon black. The industrial development of the pyrrole methodology and, in particular, of serinol pyrrole has been recently announced by a major player in the tire industry [42].

## 2. Materials and Methods

### 2.1. Materials

#### 2.1.1. Chemicals

Acetone, 2,5-hexanedione, and 2-amino-1,3-propanediol were from Sigma-Aldrich. They were used without purification.

The following chemicals were used for the preparation of the elastomeric composites discussed in this paper: TESPT (3,3′-bis(triethoxysilylpropyl)tetrasulfide from Flexys, Ann Arbor, Michigan), TDAE (H&R Olwerke Schindler GmbH, Hamburg, Germany), RIOWAX BM01 (SER S.p.A., Torino, Italy), ZnO (Zincol Ossidi, Bellusco, MB, Italy), stearic acid (Sogis, Milan, Italy), 1,3-dimethyl butyl)-*N*’-Phenyl-p-phenylenediamine (6PPD from Eastman, Kingsport, TN, USA), sulfur (S from Solfotecnica, Cotignola, Italy), *N*-tert-butyl-2-benzothiazyl sulfenamide (TBBS from Lanxess Chemical, Shangai, China), and *N*-(Cyclohexylthio)phthalimide (PVI, Brenntag, S.p.A., Milan, Italy).

#### 2.1.2. Elastomers 

Poly(1,4-*cis*-isoprene) was from Hevea brasiliensis (NR) (EQR-E.Q. Rubber, BR-THAI, Eastern GR. Thailand–Chonburi), the trade name was SIR20, and Mooney viscosity (ML (1 + 4)100 °C) was equal to 73 Mooney units (MUs). Solution Styrene Butadiene, not functionalized (SPRINTAN™ SLR 4630 from Trinseo, Milano, Italy), was used. The chemical composition of the S-SBR was: Styrene 25%, Butadiene 75%, and TDAE 37.5 phr. The butadiene comonomer had a vinyl content of 63%. Other properties: Tg = −29 °C; Mooney viscosity (ML (1 + 4) 100 °C) = 55 MU.

#### 2.1.3. Fillers

Carbon black N234 was provided by Birla Carbon (America, USA, Atlanta). The following data were reported in the technical data sheet: Oil absorption number (OAN): 125 mL/1000, nitrogen specific surface area (NSA): 119 m^2^/g, and statistical thickness surface area (STSA): 112 m^2^/g.

White micropearls of silica ZEOSIL 1165MP were from Solvay. The data in the technical data sheet were as follows: Specific surface area: 140–180 m^2^/g, loss on drying (2 h @ 105 °C) ≤ 8.0%, and soluble salts (as Na_2_SO_4_) ≤ 2.0%. In this work, the surface area was determined by using the BET method. Samples were evacuated at 200 °C for 2 h and N_2_ adsorption isotherms were recorded at 77 K in a liquid nitrogen bath by using a MICROACTIVE TRISTAR ^®^ II PLUS apparatus (Micromeritics Instrument Corporation, Norcross, GA, USA). The specific surface area (SSA) was 160 m^2^/g.

### 2.2. Preparation of SP and CB/SP Adduct

#### 2.2.1. Synthesis of 2-2,5-Dimethyl-1*H*-pyrrol-1-yl-1,3-propanediol (SP)

The key points in the synthesis of SP are: the absence of solvents, to reduce the waste; the absence of acidic substances, to prevent the oligomerization of the pyrrole compound [44]; and the removal of water, to shift the equilibrium to SP. 

In a round-bottom flask, carefully washed and dried, and equipped with a condenser (used to avoid the loss of hexanedione) and a magnetic stirrer, 16.23 g of serinol (0.1781 mol) was suspended in 21 mL of 2,5-hexanedione (0.1780 mol, 1 eq); the temperature was raised to 150 °C and stirring was performed for 2 h. The condenser was then removed, to remove water from the reaction environment, and the reaction mixture was stirred for a further 30 min. The product was collected as a brown, viscous liquid without any further purification (27.73 g, 0.1639 mol). The yield was calculated by using the following expression: 100 × (weighed mass of SP)/(theoretical mass of SP). It was calculated to be about 92%. 

^1^H NMR (CDCl_3_, 400 MHz); δ (ppm) = 2.27 (s, 6H), 3.99 (m, 4H); 4.42 (quintet, 1H); 5.79 (s, 2H). ^13^C NMR (DMSO-*d*_6_, 100 MHz); δ (ppm) = 127.7, 105.9, 43.72, 71.6, 61.2, 13.9.

#### 2.2.2. Preparation of Adducts of CB N234 with SP

The objective was the preparation of a covalent adduct of CB with SP. As mentioned in the introduction, covalent bonds are formed though a domino reaction, which begins with the oxidation of the pyrrole compound. Hence, a key point of the preparation is the presence of oxygen, which is obtained by continuously fluxing air in the reaction flask.

A total of 15 g of SP and 200 mL of acetone was poured in a 250 mL round-bottom flask. The mixture was sonicated for 5 min to promote the complete solubilization of the pyrrole compound in the solvent. In a 1 L round-bottom flask, 150 g of Pristine CB N234 was weighted. The solution of SP and acetone was poured in the 1 L round-bottom flask and 300 mL of pure acetone was added. This was made to completely cover the powder of CB N234. The mixture was sonicated for 20 min. The solvent was then removed under reduced pressure at 40 °C and the resulting dry powder was poured into an aluminum backing tray and put in a preheated oven, at 160 °C for 2 h, to perform the functionalization reaction. 

After the reaction, the CB-SP adduct was allowed to reach room temperature.

### 2.3. Preparation of Elastomer Composites

#### Elastomer Composite

**Figure 2:**
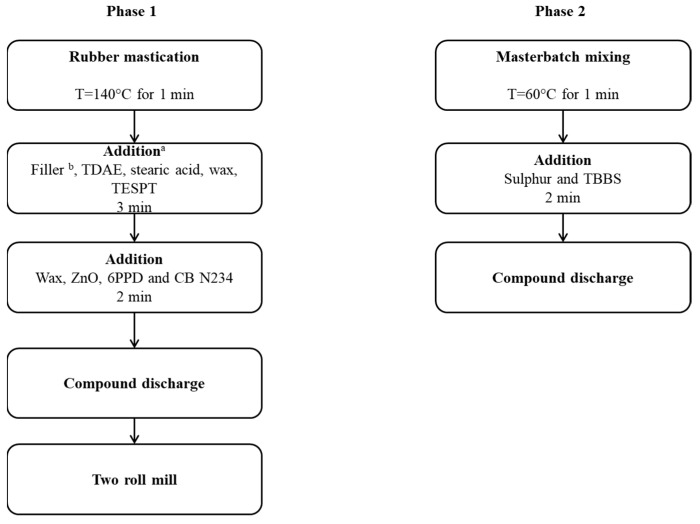
Block scheme of the processing procedure for the composites of Table 1. (a) for details see text. (b) Filler: Silica or CB N234 or CB/SP.

Formulations are shown in Table 1. The quantities of ingredients are expressed in parts per hundred rubber (phr). The reference composite was a composite with silica as the only filler. Silica was completely substituted either with CBN234 or with CB/SP. The amount of CB was calculated to have the same volume fraction of silica. The amounts of the other ingredients were the same in all the composites. The processing procedure is shown in Figure 2.

The mixing was performed in a Brabender^®^-type internal mixer with a chamber volume of 55 cm^3^. The so-called non-productive mixing was performed as follows: The rubber, reduced into crumbs, was introduced into the mixer, the temperature was brought to 140 °C, and mastication was performed for 1 min, with rotors rotating at 60 rpm. The filler and half of the amount of ingredients, such as TDAE, stearic acid, wax, and TESPT, was added, performing the mixing for 1.5 min at 140 °C. The remaining amount of the same ingredients was then added, and mixing was carried out for a further 1.5 min, at the same temperature. Ingredients such as wax, ZnO, 6PPD, and CBN234 were then added, and mixing was performed at the same temperature for 2 min. The composite discharged from the internal mixer was passed 5 times through a two-roll mill operating at 50 °C, with the front roll rotating at 30 rpm and the back roll rotating at 38 rpm, with a 1 cm nip between the rolls. The so-called productive mixing was performed as follows: The masterbatch obtained from the non-productive mixing was fed to the internal mixer at 60 °C and mixed for 1 min. The vulcanization ingredients, sulfur and TBBS, were added and mixing was performed for a further 2 min. The composites were discharged and finally homogenized by passing them 5 times through a two-roll mill operating at nominal room temperature, with the front roll rotating at 30 rpm and the back roll rotating at 38 rpm, with a 1 cm nip between the rolls. 

### 2.4. Characterization Techniques

#### 2.4.1. Thermogravimetry Analysis (TGA) of CB/SP Adducts

Thermogravimetry analyses were performed with a TGA TA instrument Q500. The following method was adoperated: heating under air flux from 30 °C to 300 °C at 10 °C/min, isothermal step at 300 °C for 15 min, heating to 550 °C at 20 °C/min, isothermal step for 15 min, heating to 900 °C at 10 °C/min, isothermal step for 3 min, and final isothermal step for 30 min. In this study, the amount of SP in the CB/SP adduct is expressed in parts per hundred carbon (phc), which are estimated by using Equation (1):

(1) $$phc=100\cdot \frac{x}{\left(100-x\right)}$$

where *x* = (weight loss)_150 °C–900 °C._

The yield of the functionalization process, *Y_f_*, from the weight of reactants to the estimation of the mass loss of the CB/SP adduct via TGA was calculated by means of Equation (2):

(2) $$Y_{f}(\%)=100\;\cdot \frac{{ml}_{w}}{{ml}_{uw}}$$

The SP mass % in CB/SP adducts after washing is *ml_w_*, while the SP mass % in CB/SP before washing is *ml_uw_*.

#### 2.4.2. Brunauer–Emmett–Teller (BET)

Specific surface area analyses were carried out via the Micromeritics TriStar 3000 instrument. Nitrogen was used as the gas and the method used followed ASTM 6556 [45].

Total surface area (NSA: nitrogen surface area) was determined in the 0.05 < p/p_0_ < 0.2 range, while the external surface area (STSA: specific thickness surface area) was determined in the p/p_0_ range from 0.2 to 0.5.

#### 2.4.3. Crosslinking

A rubber process analyzer (Monsanto R.P.A. 2000, Alpha Technologies Hudson, Hudson, OH, USA) was adoperated to perform crosslinking. A total of 5 g of rubber composite was weighed and put in the rheometer. Measurements were carried out at a frequency of 1.7 Hz and an oscillation angle of 6.98%. The sample, loaded at 50 °C, underwent a first strain sweep (0.2–25% strain) to cancel the thermo-mechanical history of the rubber composite; it was then maintained at 50 °C for 10 min and then underwent another strain sweep at 50 °C to measure the dynamic–mechanical properties at low deformations of the uncured sample. The crosslinking reaction was then carried out at 170 °C for 10 min. A torque–time curve was created. The minimum achievable torque (M_L_), the maximum achievable torque (M_H_), the time required to achieve a torque value equal to M_L_ + 1 (t_S1_), and the time required to reach 90% of the maximum torque (t_90_) were measured. 

#### 2.4.4. Structure of the Crosslinking Network

Following the procedure described in an article by the same authors [46], the samples were swollen in n-heptane for two days in a nitrogen atmosphere to allow the diffusion of the reagents. The n-heptane was then removed and the samples were washed with light petroleum and dried overnight at room temperature under reduced pressure. The samples were then immersed in toluene (200 mL) and placed in a glass tube under nitrogen. The tube was then sealed and left in the dark for 72 h, the time required for equilibrium swelling to be achieved. After this time, the samples were dried by blotting with filter paper. Finally, the samples were quickly sealed in a clean container and weighed. The samples were then dried under vacuum at 70 °C for 24 h to remove the solvent and weighed again in the dried state. This provides the amount of sorbed solvent and the weight of the dry network.

To calculate the crosslinking network, we used the Flory–Rehner equation (Equation (3)):

(3) νe=−[ln⁡1−Vr+Vr+χ1Vr2]V1(Vr13−Vr)2

where *ν_e_* is defined as the effective number of chains in a real network per unit volume; *χ*_1_ is the polymer solvent interaction parameter; and *V*_1_ is the molecular volume of the solvent. *Vr* is defined as the volume fraction of the polymer in a swollen network in equilibrium with pure solvent. The mathematical definition is reported in Equation (4):

(4) $$V_{r}=\frac{\frac{m_{dr}}{\rho_{dr}}}{\frac{m_{dr}}{\rho_{dr}}+\frac{m_{s}}{\rho_{s}}}=\frac{V_{dr}}{V_{dr}+V_{s}}$$

where *m_dr_* is the weight of dry rubber, *ρ_dr_* the density of dry rubber, *m_s_ is* the weight of the absorbed solvent, and *ρ_s_* is the density of the solvent. For the mono- and di-sulfidic crosslink measurements, the procedure followed was very similar. 

A total of 100 mg of crosslinked composite was placed in a 20 mL beaker with 100 mL of heptane. The sample was left to stand for 24 h. After this time, 3.8 mL of propanethiol and 4 mL of piperidine were added, and the mixture was left at room temperature for 2 h. The mixture was then washed 3 times in 50 mL of heptane and then filtered. The solid was left in heptane for a further 24 h and then washed in light petroleum. The sample was filtered under vacuum and dried under reduced pressure for 24 h. The procedure was identical to that described above. The correction for the presence of the filler was taken into account.

#### 2.4.5. Dynamic–Mechanical Analysis in the Shear Mode: The Strain Sweep Test

The shear dynamic–mechanical characteristics of rubber composites were evaluated by performing strain sweep tests in a rubber process analyzer (Monsanto R.P.A. 2000). As reported in Section 2.4.3, the crude sample was subjected to a first strain sweep, then held at 50 °C for ten minutes, followed by another strain sweep at 50 °C. Data from the second strain sweep were collected and are reported in the text below to discuss the behavior of uncured samples. The crosslinking was then carried out, as reported in Section 2.4.4, and the shear dynamic–mechanical properties of the cured samples were then assessed after 20 min at 50 °C using a 0.2–25% strain sweep at a frequency of 1 Hz. Shear storage and loss moduli (G′, G″), and subsequently Tan δ, were measured characteristics.

All the tests performed with the rubber process analyzer were kept under quality control, determining the repeatability and the reproducibility of the measurements [47]. Data reported in the manuscript were characterized by a variation coefficient in the range from 0.5% to 1%. 

#### 2.4.6. Dynamic–Mechanical Analysis in the Axial Mode

The elastomeric compound was rolled up to obtain a long cylinder. This cylinder was then cut into smaller cylinders and vulcanized (at 170 °C for 20 min) to produce cylindrical test pieces with dimensions of 25 mm in length and 12 mm in diameter. An Instron dynamic device in the traction–compression mode was employed to perform dynamic mechanical measurements, and was maintained at the predetermined temperatures (10, 23, and 70 °C) throughout the entire experiment. The cylinder was pre-loaded to a 25% longitudinal deformation with respect to the original length. The compression was subjected to a dynamic sinusoidal strain in compression with an amplitude of around 3.5% regarding the length under pre-load, at a frequency of 100 Hz. The values of the dynamic storage modulus (E′), loss modulus (E″) and loss factor (tan δ) were calculated as the ratio between E″ and E′.

All the tests performed with the rubber process analyzer were kept under quality control, determining the repeatability and the reproducibility of the measurements [47]. Data reported in the manuscript were characterized by a variation coefficient in the range from 0.5% to 1%.

#### 2.4.7. Tensile Test

Standard dumbbells made from 10 cm by 10 cm by 1 mm vulcanized compound plates were used to perform tensile tests at room temperature with a Zwick Roell Z010 and an optical extensometer. Measurements were performed at 1 mm/min. Stresses at different elongations (σ_50_, σ_100_, and σ_300_), stress at break (σ_B_), elongation at break (ε_B_), and the energy required to break were measured according to Standard ISO 37/UNI 6065 [48].

#### 2.4.8. Digital Filler Dispersion Analysis through Transmission Electron Microscopy

The digital image analysis of filler dispersion through TEM was carried out following three steps: (1) the vulcanized compound was sliced with a diamond cryo-cutter under liquid nitrogen to obtain sections with thicknesses around 100 nm, (2) sections were observed in the transmission electron microscope and 10 images were acquired with a digital camera for each sample, and (3) the acquired images were visually analyzed to determine the amount of undispersed filler particles.

TEM characterizations were performed with a TEM, LIBRA^®^ 120, Zeiss, Oberkochen (Baden-Württemberg, Germany), with an acceleration voltage of 120 kV.

Ultra-thin sections must be prepared from the sample material in order to analyze it using the TEM. For this purpose, a cryo-ultramicrotome (type: Leica EM FC 6, Leica Microsystem, Wetzlar, Germany) using a diamond knife (type: Diatome 35°) at a temperature of −80 °C was used to produce sections with a width approx. 100 µm and a thickness approx. 100 nm. The preparation was carried out using the wet-cutting method, in which the sections are floated on a mixture of dimethyl sulphoxide (DMSO) and water (50/50) and transferred to a copper mesh using a loop. To stabilise the sections in the transmission electron microscope, they were placed on a 400 mesh copper carrier coated with poly(vinyl formal) (Plano).

#### 2.4.9. Scanning Electron Microscopy (SEM)

The SEM investigation was carried out using a Zeiss EVO 50 EP SEM (Zeis Vision Care, Castiglione Olana, Italy) coupled with an EDS spectrometer (Bruker Quantax 200 6/30, Bruker, Billerica, MA, USA). CB powders were applied on aluminum stubs with the aid of a conductive carbon bioadhesive. Although CB is already conductive, the samples were coated with gold using an Ewards S150B sputter coater (Perkin Elmer, Milan, Italy) and evaluated.

## 3. Results and Discussion

### 3.1. Preparation and Characterization of SP and CB/SP Adduct

Serinol pyrrole was prepared, as previously reported [26], by mixing 2-amino-1,3-propanediol (serinol) with 2,5-hexanedione and heating at 150 °C for 2 h 30 min, achieving a yield of 92%. Details are in the Materials and Methods Section.

**Figure 3:**
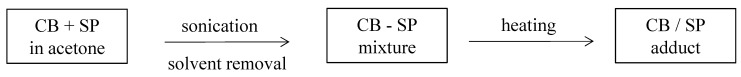
Block diagram for the preparation of the CB/SP adduct.

The CB/SP adduct was prepared as summarized in the block diagram in Figure 3.

In brief, CB and SP were mixed in acetone, and a homogenous mixture was formed via sonication. This procedure is used at the lab scale, but both the organic solvent and sonication can be avoided at the pre-industrial scale [42], where a spray dryer [49] can be used. The physical mixture, obtained via the removal of the solvent at a reduced pressure, was heated at 150 °C for 2 h. As reported in the introduction, a domino reaction occurred [27], with the formation of a covalent CB/SP adduct, bearing OH functional groups. Research is in progress to reduce the temperature of the reaction, for example, by using a pre-oxidized pyrrole compound.

#### 3.1.1. Thermogravimetric Analysis (TGA)

The amount of the organic modifier in the CB/SP adduct was estimated by means of TGA, performed on both pristine CB N234 and on the CB/SP adduct. The analyses were performed in a temperature range from 30 °C to 900 °C, with a heating rate of 10 °C/min under N_2_. A TGA traces are reported in Figures S1 and S2 in the Supplementary Materials, and the data are shown in Table 2.

The mass loss peak of SP occurred at about 250 °C, as is visible in Figure S2 in the Supplementary Materials. The loss below 150 °C can be attributed to water, considering the hydrophilic character of SP (easily soluble in water). A three-step decomposition profile was observed for the carbon materials. The first mass loss below 150 °C is usually attributed to low molar mass substances, such as water or acetone used for washing the adduct [23,25,26]. The mass loss at 150 °C < T < 900 °C can be attributed to the alkenylic defects of the carbon substrate and to the organic modifier. The carbon substrate accounts for the mass loss at T > 900 °C. The residue is due to the inorganic components. For CB/SP, the peak of the largest mass loss below 900 °C was at about 450 °C (see Figure S2 in the Supplementary Materials). A small peak was visible in the TGA trace of the unwashed CB/SP sample at about 330 °C, whereas it was absent from the trace of the washed adduct. These findings indicate that the CB/SP adduct is based on a strong interaction between the pyrrole compound and the carbon substrate, the covalent bond arising from the cycloaddition, the last step of the domino functionalization reaction [27]. The CB/SP adduct was analyzed before and after acetone washing and the content of SP in the adduct was expressed in parts per hundred carbon (phc), calculated by applying Equation (1) (see the Materials and Methods Section). In the unwashed sample, the SP content was estimated to be 10, as expected, whereas 6.8 phc of SP were found in the washed sample. This value led us to evaluate a functionalization yield of 68% by applying Equation (2) (see the Materials and Methods Section). A very low amount of residue was observed. A nominal value of 10 phc of PyC in the adduct corresponds to 108 mmol OH/100 g of adduct. In the case of the silica grade used in this study, the amount of OH groups can be calculated [17] to be 176 mmol(OH)/100 g(silica), or 44.2 mmol(OH)/100 g(silica) by considering the most reactive isolated silanols. Hence, the functionalization of CB with SP led to the introduction of at least a comparable amount of OH groups.

#### 3.1.2. Brunauer–Emmett–Teller (BET) Surface Area of CB Samples

The total surface area (NSA) and the external surface area (STSA) of pristine CBN234 and washed CB/SP adduct were determined by means of nitrogen adsorption measurements, performed with the BET technique. Data are shown in Table 3.

The functionalization with SP led to an appreciable reduction in both the total and the specific surface areas.

#### 3.1.3. Scanning Electron Microscopy

**Figure 4:**
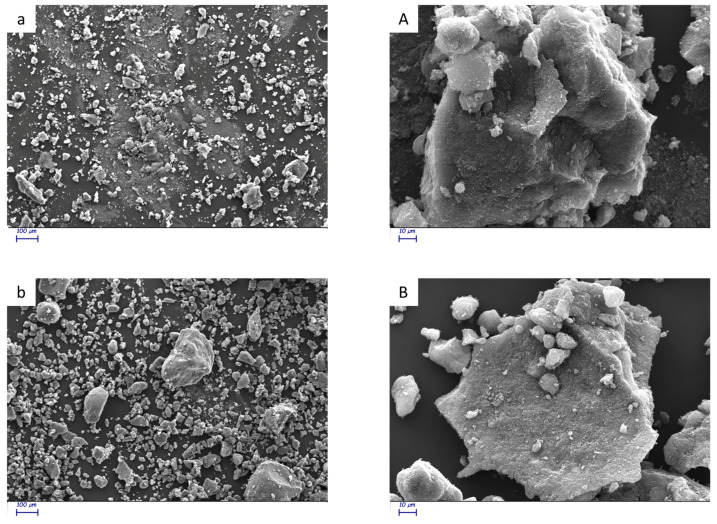
SEM images at lower (250×) and higher magnitudes (2000×). Pristine CB: (**a**) lower magnitude, (**A**) higher magnitude; CB/SP: (**b**) lower magnitude, (**B**) higher magnitude.

The SEM investigation was carried out on gold-sputtered samples of CB and CB/SP, at different magnifications (250× and 2000×). Sixteen micrographs were taken for each sample and representative images were selected and are visible in Figure 4. Pristine CB images are shown in Figure 4a for the lower magnification and in Figure 4A for the higher magnification; CB/SP images are presented in Figure 4b for the lower magnification and in Figure 4B for the higher magnification.

The images reveal the higher distribution of aggregate sizes for CB/SP, which shows small but also large aggregates (up to more than 280 microns), compared to pristine CB (maximum size of about 80 microns). It can be said that the OH on the CB surface favors the aggregation of CB particles.

### 3.2. Elastomer Composites with the CB/SP Adduct

The unwashed CB/SP adduct was used for the preparation of the elastomer composite. The selection of the unwashed sample was made in view of possible industrial development; indeed, the adduct would not be washed in an industrial process. A traditional formula for a tire-tread compound was used, with S-SBR and NR as the elastomers and 70 phr of silica in the reference silica composite. CB and CB/SP adduct were used at the same volume fraction, and this allowed us to reduce the density of the compound from 1.21 g/cm^3^ to 1.15 g/cm^3^.

#### 3.2.1. Crosslinking

**Figure 5:**
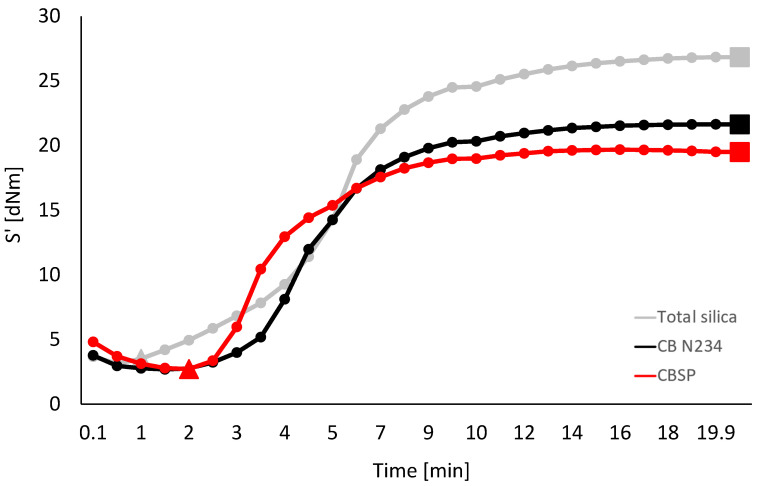
Torque vs. time measured during the crosslinking of the composites in Table 1. The triangles identify the experimental value of M_L_; the squares identify the experimental value of M_H_.

Sulfur-based crosslinking was performed at 170 °C for 20 min. Values of minimum modulus, M_L_, maximum modulus, M_H_, induction crosslinking time, t_s1_, optimum crosslinking time, t_90_, and curing rate are visible in Table S1 in the Supplementary Materials. The crosslinking curves are shown in Figure 5.

A higher M_L_ value was obtained for the silica composite, as expected, considering that M_L_ is an index of the viscosity of the composite. In the presence of composites with the same elastomer matrix, the same filler volume fraction, and the same amount of so-called small ingredients, different M_L_ values should be ascribed to the extent of the filler network, which is reasonably higher in the presence of a filler with pronounced supramolecular interactions, such as silica. The M_H_ value is due to both the crosslinking and the filler networks. Indeed, the amplitude achieved in the shear test (see the Materials and Methods Section) is not sufficient to completely remove the filler network. The M_H_ and (M_H_ − M_L_) values were remarkably higher for the silica composite. It is worth commenting on the differences between the CB and the CB/SP composite. Faster curing was obtained with the CB composites, as expected. CB/SP produced the lowest values for induction and optimum time of vulcanization and the highest value for curing rate. These findings are in line with what has already been reported [26] and could be due to sp^3^ nitrogen atoms on the CB surface.

The structure of the crosslinking network was studied by performing swelling experiments, as explained in the Materials and Methods Section. Values of total crosslinks, mono- and di-sulphides, and poly-sulphides are presented in Table 4.

The same values were obtained for the silica and the CB/SP composites, whereas a higher number of total crosslinks was achieved with the CB composite. In a previous study by some of the authors [46], it was shown that the total number of crosslinks increased with the surface area of the sp^2^ carbon allotrope. Differences in the distribution of sulfur bridge lengths among the three composites cannot really be appreciated, though the higher amount of mono- and di-sulfides in the case of CB-SP could also be attributed to the presence of the basic nitrogen on the CB surface, which is expected to interact with the sulfur chains.

#### 3.2.2. Shear Dynamic–Mechanical Properties

Dynamic–mechanical properties were determined in the shear mode by applying the procedure described in the Materials and Methods Section, exploring a strain amplitude ranging from 0.2% to 25% and measuring the storage (G′) modulus and the loss (G″) modulus. Values of G′_γmin_, G′_γmax_, ΔG′, ΔG′/G′_γmin_, G″_max_, and Tanδ_max_ (Tan delta = G″/G′) are reported in Table 5, while G′ vs. strain and Tan delta vs. strain curves are shown in Figure S3 and Figure S4, respectively, in the Supplementary Materials.

Lower values of G′_γmin_ and ΔG′ were obtained with the CB composites, in particular with the CB/SP composite. The values of ΔG′/G′_γmin_ appear to be similar for the three composites. ΔG′ and ΔG′/G′_γmin_ values provide indication of the Payne effect [47,48,49], which is essentially due to the filler networking phenomenon and causes the dissipation of energy. The higher values of G′_γmax_ for the silica composite appear to be in line with the higher M_H_ value. The tanδ_max_ values are similar for the silica and the CB/SP composites, whereas they are is appreciably higher for the CB composite. These results suggest that the functionalization of CB with SP does play a role, favoring the reduction in the filler networking phenomenon, whose result is definitely not due to the presence of TESPT alone. The lower Tan delta values are also in line with the slightly better dispersion of CB/SP in the rubber matrix, documented by TEM analysis.

#### 3.2.3. Dynamic–Mechanical Properties in the Axial Mode

**Figure 6:**
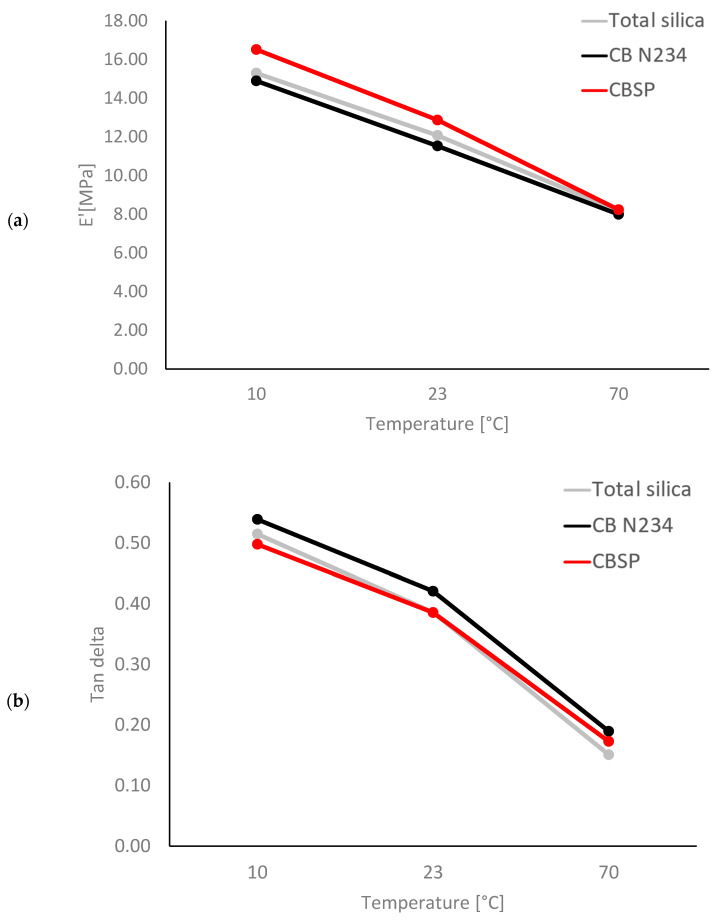
Storage modulus E′ vs. temperature (**a**) and Tan delta vs. temperature (**b**) for composites in Table 1.

Axial dynamic–mechanical properties were measured in compression, as described in the Material and Methods Section, determining E′, E″, and Tan delta (E″/E′) values at 10 °C, 23 °C, and 70 °C, respectively. The data are presented in Table 6, and the values at the different temperatures of E′ and Tan delta are plotted vs. the temperature in Figure 6 (Figure 6a and Figure 6b, respectively).

The composite with CB/SP revealed the highest dynamic rigidity at all the temperatures. The CB composite has the lowest dynamic rigidity and the highest hysteresis. The procedure adopted for the measurements allows us to exclude the influence of the filler network. Indeed, a pre-strain was applied to remove the interactions involving filler aggregates. Moreover, the higher E′ values obtained with CB/SP compared with CB cannot be attributed to the total crosslinks, which are higher for the latter composite. Hence, the system formed by CB/SP + TESPT appears capable of effectively reinforcing the elastomer matrix, establishing a stable interaction between CB and the elastomer chains. The Tan delta values, which are an indication of the composite’s hysteresis, are similar for the silica and the CB/SP composites. However, a crossover of the lines due to silica and to CB/SP can be observed in Figure 6. It is known that a silica-based composite, compared to a CB-based composite, has lower hysteresis at a high temperature (low frequency) and similar/higher hysteresis at a low temperature (high frequency). This is typical of silica-based composites and promotes road traction and low rolling resistance when the composites are used in tire treads. This behavior can be explained by the chemical bond between silica and the elastomer chains that arises from the reaction of silanols with silane. As reported above, the amount of OH on the CB surface was 108 mmoles/100 g of CB/SP adduct, an amount which is at least comparable to that of the OH groups in silica. The slightly different dependence of hysteresis on the temperature could be explained by the different reactivity of the OH groups from SP compared with silanols. It is worth mentioning that the highest dynamic rigidity would allow us to reduce the amount of filler in the CB/SP composite, thus obtaining a reduction in hysteresis. It is worth highlighting the difference between the CB and CB/SP composites. Also, these results indicate that CB/SP is able to interact with TESPT and then with elastomer chains.

#### 3.2.4. Tensile Properties

Tensile properties were measured by means of quasi-static measurements, measuring the stresses σ_50%_ σ_100%_ σ_200%_ σ_300%_ σ_break_, the elongation at break, Ɛ_break_, and the energy at break. The data are reported in Table 7 and the stress–strain curves are presented in Figure S5 in the Supplementary Materials.

The silica composite reveals the highest stresses at all the strains and the best ultimate properties. The tensile properties of the CB/SP composite are not that far off and are better than those of the CB composite. These findings confirm that the CB/SP + TESPT system can promote the reinforcement of an elastomer matrix. They also suggest that the formulation of the composite should be properly tuned, as this has been performed in recent decades for silica-based composites.

#### 3.2.5. Transmission Electron Microscopy for the Distribution of Filler Analysis

**Figure 7:**
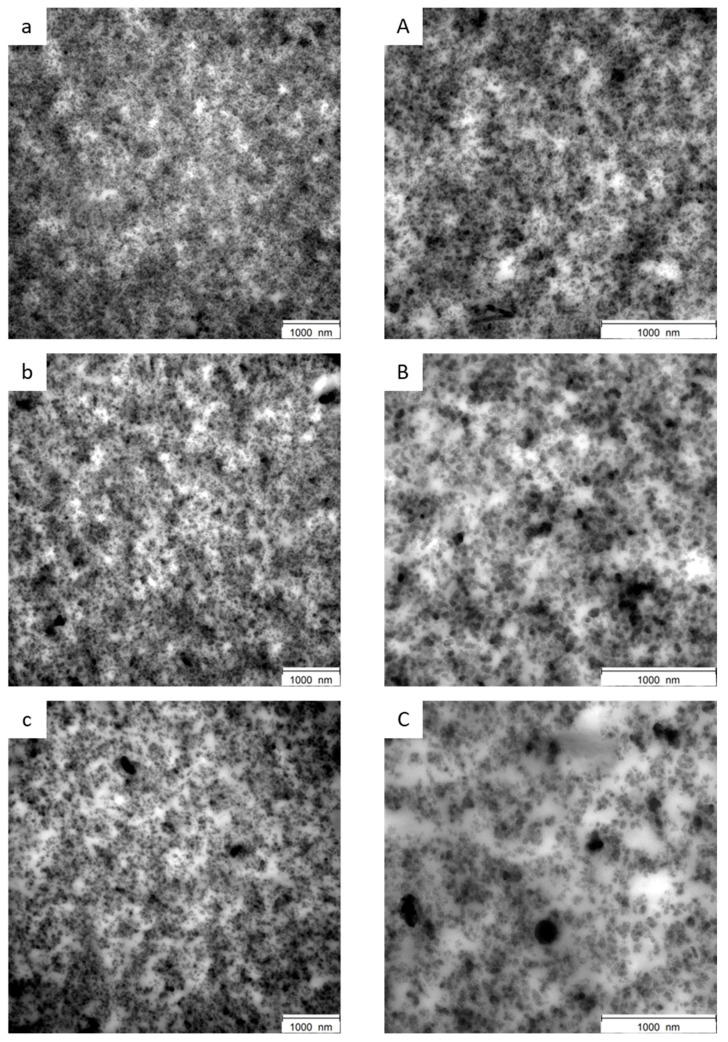
TEM images at lower and higher magnitudes. Silica (**a**) lower magnitude, (**A**) higher magnitude; CBN234: (**b**) lower magnitude, (**B**) higher magnitude; CB/SP: (**c**) lower magnitude, (**C**) higher magnitude.

Transmission electron microscopy (TEM) analysis was performed on the three vulcanized composites, with the aim to investigate the filler dispersion, 12 micrographs were taken for each composite, at low and high magnifications, and representative images were selected. TEM micrographs can be seen in Figure 7 for the silica composite (Figure 7a,A), the CB composite (Figure 7b,B), and the CB/SP composite (Figure 7c,C).

According to the formula of the three compounds in all images, the two phases of the polymer can be observed. The light phase (white to light gray) corresponds to NR, whereas the dark-gray phase is to be assigned to S-SBR. The black agglomerates of CB are distributed in the direction of the SBR phase, which can be explained by the chemical compatibility of CB with the aromatic systems in the SBR (Figure 7b,B,c,C). Furthermore, an even distribution of small aggregates, with a very low amount of agglomerates as well primary particles (size of appr. 25–35 nm) and absence of flaws can be observed for the silica and the CB composites, both at high and low magnifications. In addition, the micrographs of the CB/SP composite material show the presence of a small number of large particles (size of appr. 250 nm), which are not part of the filler; nevertheless, a clear identification cannot be made from the image itself (Figure 7c,C). The TEM images show only a small part of the sample in two dimensions (2D). But, overall, small particles with a size of approximately 30 nm are well dispersed in the SBR phase (dark gray). A lower ΔG′ was observed for the CB/SP system in comparison with the others in the amplitude sweep experiments (Table 4 and Figure S3 in the Supplementary Materials). This finding is in line with the slightly better dispersion shown in the TEM image (Figure 7).

#### 3.2.6. Comparison of the Behavior of CB/PyC Adducts in Place of Silica

As reported in the introduction, CB/PyC adducts with PyC containing sulfur atoms were used as reinforcing fillers in elastomer composites based on S-SBR and NR [24,29]. The objective in common with the work reported in this manuscript is the preparation of composites with dynamic–mechanical properties similar to those of a silica-based compound, hence suitable for a tire tread. It is worthwhile to compare the results obtained with the two families of pyrrole compounds. Normalized data are shown in Table S2 in the Supplementary Materials: the composite based on pristine CB was taken as a reference and the relative values of dynamic–mechanical (in the shear and axial modes) and tensile properties were calculated.

Compared with the composite with pristine CB, the PyC grafted on CB led to more efficient curing (lower induction time and higher curing rate), to the reduction in ΔG′, to higher dynamic rigidity (E′ values at both 10 °C and 70 °C), and to lower hysteresis at 70 °C. The PyC with sulfur atoms appeared to be more efficient in promoting these effects. The CB/SP adduct led to lower stresses at different elongations and to similar stresses with a larger elongation at break. Similar tensile properties were obtained with SHP and SSP with somewhat lower values with the former PyC. These comparisons confirm that CB bearing functional groups reactive with the elastomer chain, either directly or through a coupling agent, create to composites with improved dynamic–mechanical reinforcement and lower hysteresis. The direct reactivity, promoted by the sulfur atoms, appears to be more efficient.

## 4. Conclusions

This work demonstrates that silica is not the only filler for reinforcing elastomer composites for dynamic mechanical applications with low energy dissipation. CBN234 functionalized with serinol pyrrole and thus bearing hydroxy functional groups was used in place of silica, at the same volume fraction, and the same coupling agent traditionally employed for silica, silane TESPT, was capable of promoting the interaction of CB/SP with the elastomer chains. The composite with the CB/SP + TESPT system shows better dynamic–mechanical properties than the composite with pristine CB + TESPT, despite the lower number of total crosslinks. The dynamic rigidity increased by 11%, the hysteresis was reduced by 11%, the stress at break decreased by 2%, and elongation at break increased by 11% and 36%. The properties of the silica and the CB/SP composites are comparable. Differences, such as the higher dynamic rigidity at a low temperature, and the poorer ultimate tensile properties could be ascribed to the worse filler dispersion. Indeed, CB/SP is a polar filler, and appropriate processing and composite environments, e.g., with suitable covering agents, should be developed. The use of CB in place of silica allows the preparation of lighter (5% in the present study), electrically conductive compounds.

This study demonstrates that the dynamic–mechanical reinforcement with low hysteresis of an elastomer composite is promoted by a high-surface-area nanostructured filler that can establish a chemical bond with elastomer chains. To date, this behavior is a monopoly of silica. The significance of the results reported here lies in demonstrating the role of filler features, and, in particular, of the chemical reactivity of the filer surface, regardless of the nature of the filler. This paves the way for the development of circular fillers, such as the chars coming from biosources and from end-of-life tires.

## Figures and Tables

**Table 1:** Formulation of S-SBR/NR-based composites with CB/SP.

Ingredient ^a^	Silica	CB N234	CB/SP
S-SBR 4630	70	70	70
NR (SIR—20)	30	30	30
TESPT	5.2	5.2	5.2
Silica	70	0	0
CB N234	5.2	65.2	5.2
CB/SP	0	0	60

^a^ Other ingredients (phr): TDAE 5, riowax BM01 1, stearic acid 2, ZnO 2.5, 6PPD 2, sulfur 2, TBBS 1.8, and N-cyclohexylthiophthalimide 0.5.

**Table 2:** Mass losses of pristine CB and CB/SP adduct from TGA.

Sample		Temperature Range				Amount of SP (phc)
	T < 150 °C	150 °C < T < 550 °C	550 < T < 900 °C	T > 900 °C	Residue	
SP	4.3	93.9	0.3	0.3	2	
CB N234	0.8	1.2	1.1	96.7	0.2	-
CB/—P—UW ^a^	0.4	7.2	1.9	90.1	0.4	10
CB/—P—W ^b^	0.5	5.3	1.7	90.6	1.9	6.8

^a^ UW: unwashed. Before acetone extraction; ^b^ W: washed. After acetone extraction.

**Table 3:** BET total and external surface areas for pristine CB and CB/SP.

Sample	Total Surface Area (NSA) [m^2^/g]	External Surface Area (STSA) [m^2^/g]
CBN234	112	108.8
CB/SP	86.2	71.0

**Table 4:** Structure of the crosslinking network of composites in Table 1.

Composites			
	Silica	CB	CB/SP
Total crosslinks (mol/g)	3.24	4.92	3.29
Mono- and di-sulphides (% mass)	47.90	46.10	47.90
Poly-sulphides (% mass)	52.10	53.90	52.10

**Table 5:** Dynamic–mechanical properties of S-SBR4630 compounds obtained through strain sweep experiments.

	Silica	CB	CB/SP
**G′_γmin_ [Mpa]**	3.93	3.62	3.35
**G′_γmax_ [MPa]**	1.66	1.38	1.27
**ΔG′ ^a^ [MPa]**	2.27	2.24	2.07
** $\Delta G\prime /G_{\gamma_{min}}^{\prime }$ **	0.58	0.62	0.62
**G″_max_ [MPa]**	0.49	0.56	0.39
**tanδ_max_**	0.18	0.24	0.20

^a^ ΔG′ = G′_γm—n_ − G′_γmax._

**Table 6:** Axial dynamic–mechanical properties in the axial mode of composites in Table 1.

	T [°C]	Silica	CB	CB/SP
**E′ [MPa]**	10	15.30	14.89	16.52
	23	12.08	11.53	12.87
	70	8.17	7.99	8.24
**E″ [MPa]**	10	7.87	8.02	8.22
	23	4.66	4.85	4.96
	70	1.23	1.52	1.42
**Tanδ**	10	0.52	0.54	0.50
	23	0.39	0.42	0.39
	70	0.15	0.19	0.17

**Table 7:** Tensile properties of S-SBR 4630 compounds obtained through stress–strain experiments.

	Composite		
	Silica	CB	CB/SP
**σ_50%_ [MPa]**	2.19	2.29	2.04
**σ_100%_ [MPa]**	3.83	4.74	3.61
**σ_200%_ [MPa]**	9.60	12.86	8.71
**σ_300%_ [MPa]**	17.08	-	14.50
**σ_300%_/σ_100%_**	4.45	-	4.01
**σ_break_ [MPa]**	18.53	15.37	15.13
**Ɛ_break_ [%]**	320.94	229.79	313.30
**Energy break [MJ/m³]**	25.71	15.23	21.66

## Data Availability

Data are contained within article and Supplementary Materials.

## Supplementary Materials

The following supporting information can be downloaded at: https://www.mdpi.com/article/10.3390/polym16091214/s1, Figure S1: Thermograph from TGA analysis of CB/SP, Figure S2: Thermogravimetric curves for SP (a), CB (b), CB/SP UW (c); CB/SP W (d); Figure S3: Storage modulus curves for S-SBR 4630 compounds, Figure S4: Tan delta curves for SBR 4630 compounds, Figure S5. Tensile curves of S-SBR 4630 compounds obtained through stress strain experiments; Table S1: Data from the crosslinking of composites of Table 1; Table S2: Normalized data for curing, dynamic-mechanical (in the shear and axial modes) and tensile properties for composites with CB/PyC as the reinforcing filler ^a^.
