# Supplementary material for: Carbon Black Functionalized with Serinol Pyrrole to Replace Silica in Elastomeric Composites
Source: Polymers (Basel). 2024 Apr 26;16(9):1214. doi: 10.3390/polym16091214 (PMC11085758; doi:10.3390/polym16091214)
Supplement: Supplementary file 1 [file polymers-16-01214-s001.zip › polymers-2889195-supplementary.pdf]

Carbon Black Functionalized with Serinol Pyrrole to Replace Silica in Elastomeric Composites

Federica Magaletti [1], Martina Galbusera [1], Davide Gentile [1], Ulrich Giese [2], Vincenzina Barbera [1,*] and Maurizio Galimberti [1,*]

[1] Department of Chemistry, Materials and Chemical Engineering "G. Natta", Politecnico di Milano, Via Mancinelli 7, 20131 Milan, Italy; federica.magaletti@polimi.it (F.M.); martina.galbusera@mail.polimi.it (M.G.); davide.gentile@polimi.it (D.G.)

[2] Deutsches Institut für Kautschuktechnologie e. V., Eupener Straße 33, 30519 Hannover, Germany; ulrich.giese@dikautschuk.de

* Correspondence: vincenzina.barbera@polimi.it (V.B.); maurizio.galimberti@polimi.it (M.G.)

Keywords: carbon black; serinol pyrrole; functionalization; rubber compounds; elastomeric composites; Payne effect; hysteresis

Supplementary Material

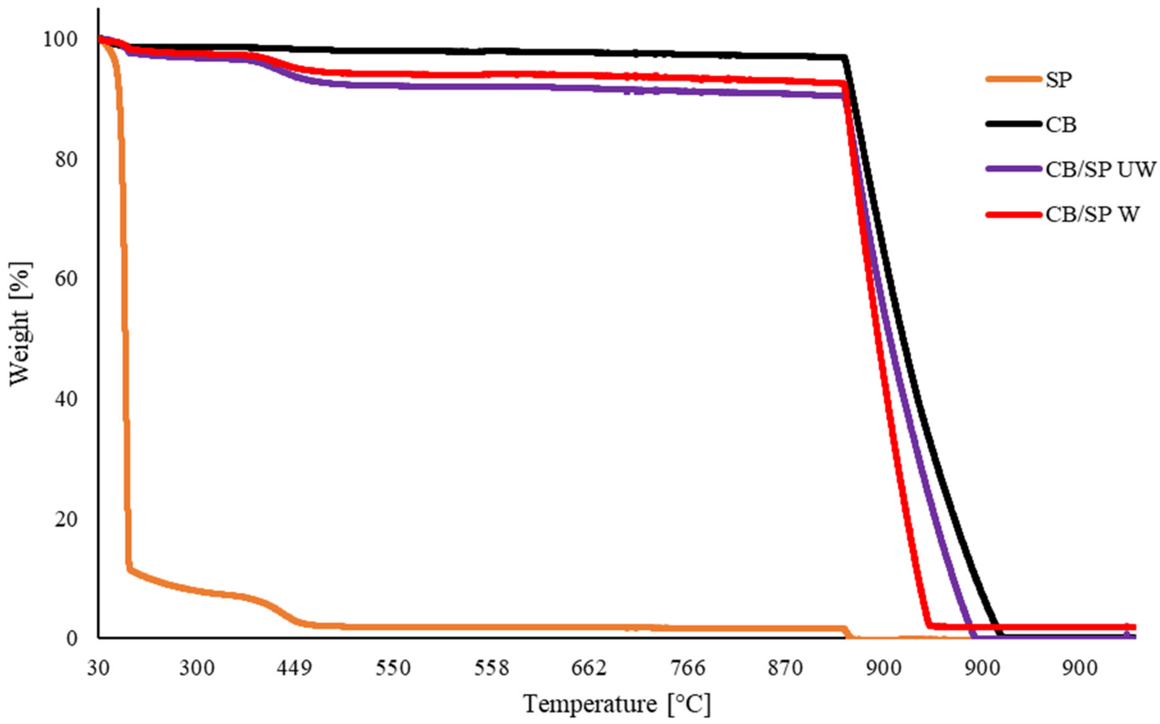

Figure S1. Thermograph from TGA analysis of CB/SP

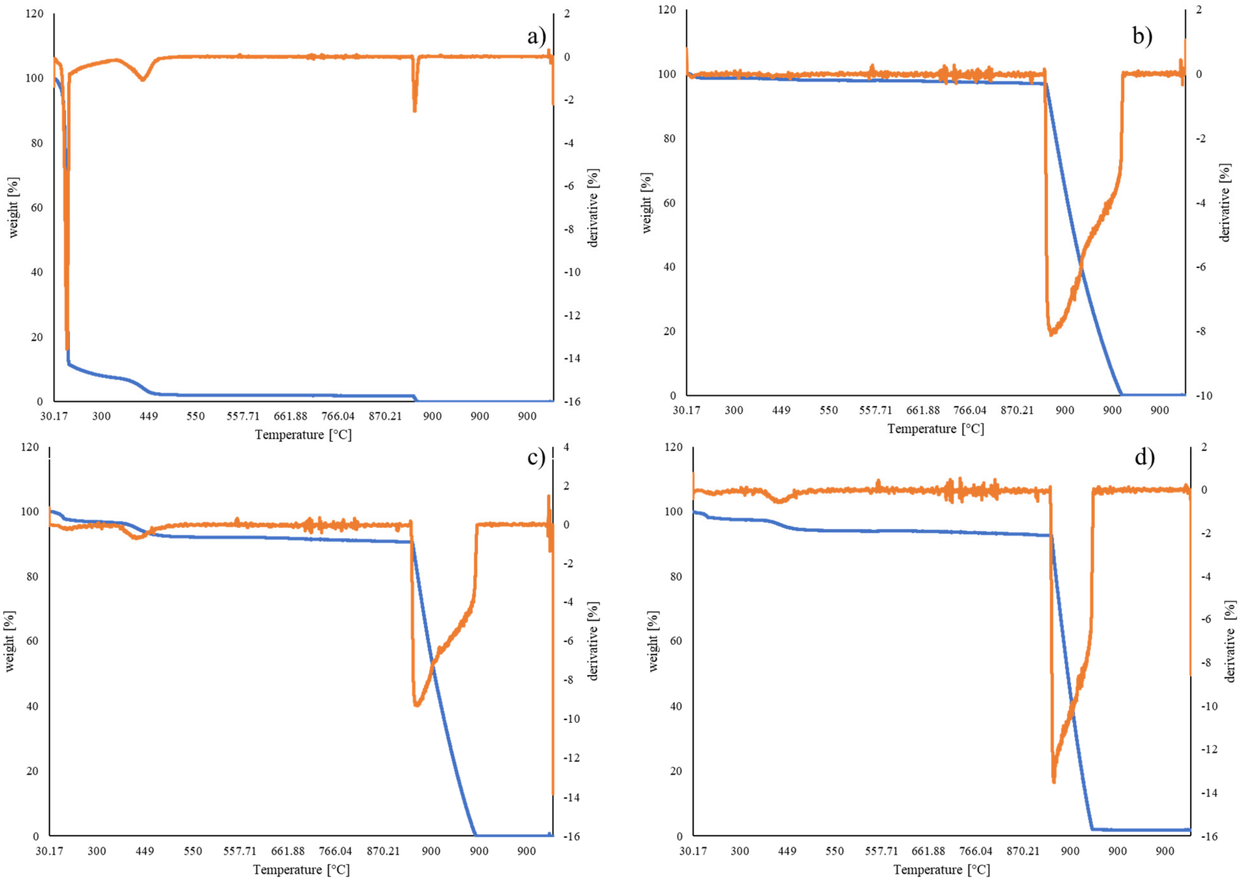

Figure S2. Thermogravimetric curves for SP (a), CB (b), CB/SP UW (c); CB/SP W (d);

Table S1. Data from the crosslinking of composites of Table 1[a,b]

	**Silica**	**CB N234**	**CB/SP**
**M_L [dNm]**	3.12	2.68	2.67
**M_H [dNm]**	26.85	21.64	19.68
**M_H-M_L [dNm]**	23.73	18.96	17.01
**t_{S1} [min]**	2.32	3.28	2.76
**t_{90} [min]**	9.88	8.96	7.61
**curing rate [dNM/min]**	3.14	3.34	3.51

[a] For experimental details see the experimental part

[b] M_L: minimum modulus, M_H: maximum modulus, t_{s1}: induction crosslinking time, t_{90}: optimum crosslinking time,

[c] the curing rate was calculated by means of the following equation:

$$Curing\ Rate = \frac{M_H - M_L}{t_{90} - t_{s1}}$$

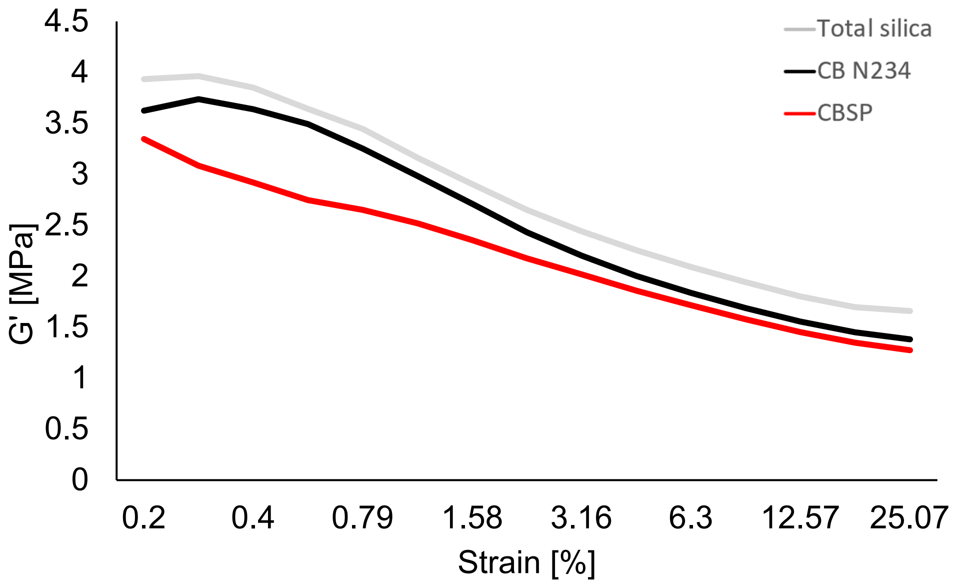

Figure S3. Storage modulus curves for S-SBR 4630 compounds.

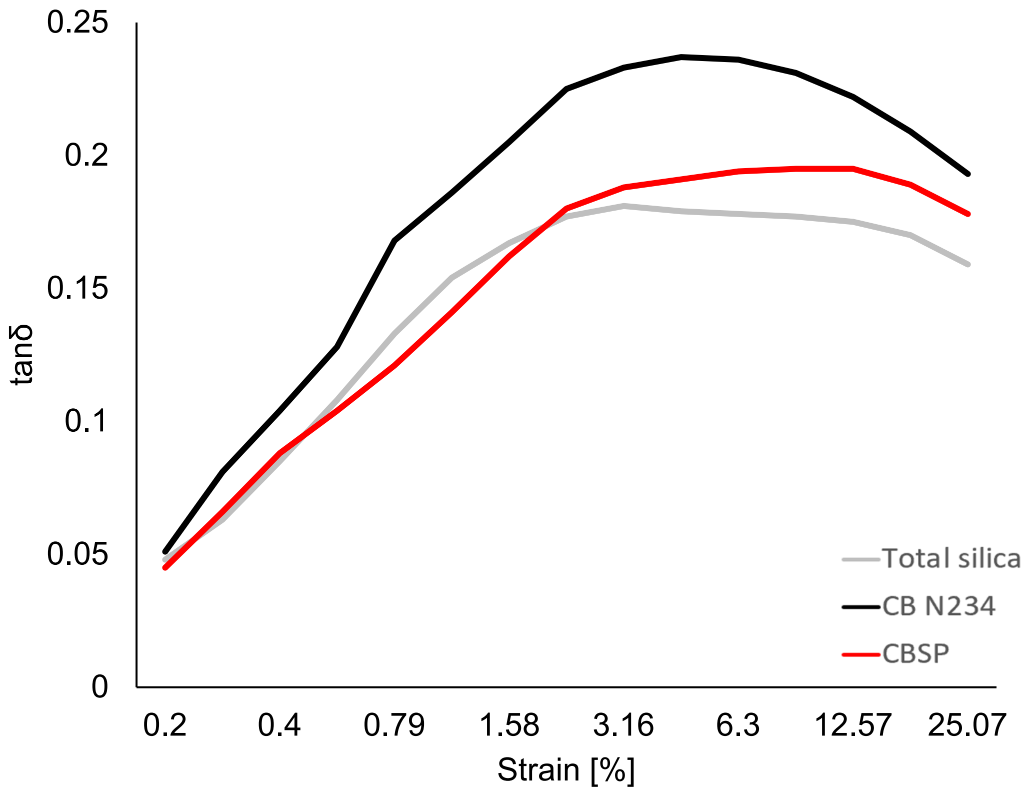

Figure S4. Tan delta curves for SBR 4630 compounds.

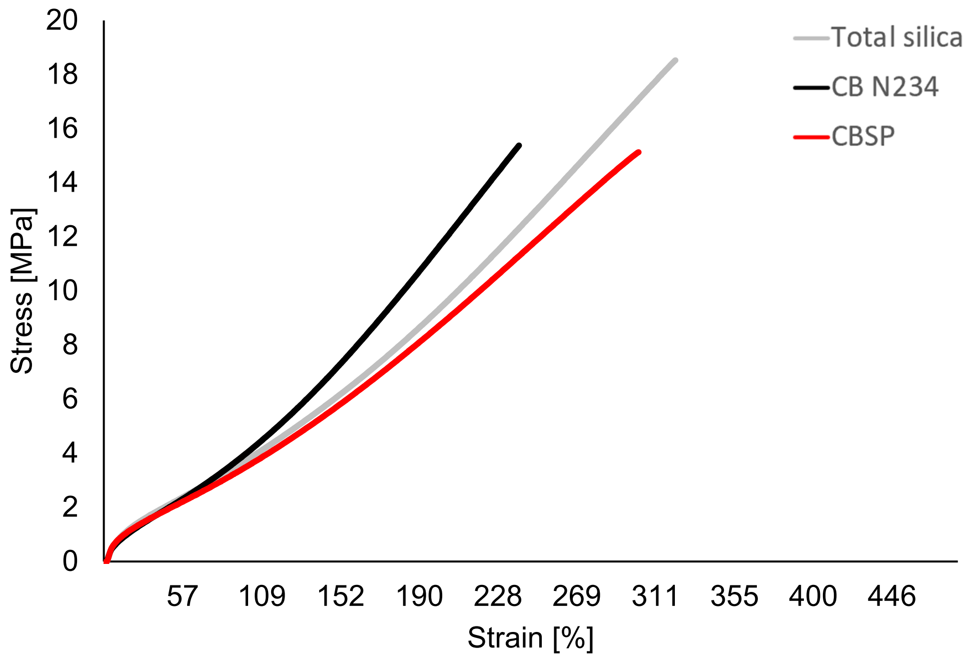

Figure S5. Tensile curves of S-SBR 4630 compounds obtained through stress strain experiments.

Table S2. Normalized data for curing, dynamic-mechanical (in the shear and axial modes) and tensile properties for composites with CB/PyC as the reinforcing filler[a].

Parameter	CB/SP[b]	CB/SHP[c] 33%	CB/SSP[d] 33%	CB/SSP[d] 66%
t_{s1}	84	94	94	91
curing rate [dNM/min]	105	137	123	140
ΔG′	92	84	99	93
E' (10°C)	111	118	111	119
E' (70°C)	103	123	112	121
Tanδ (70°C)	89	92	96	92
σ100	76	109	106	118
σ200	68	99	102	108
σ_{break}	98	87	101	101
ε_{break}	136	93	100	99

[a] The reference composite was based on pristine CB. The values of the reference composites were placed = 100.

[b] ref: this work

[c] SHP: Ref [1]

[d] SSP: ref [1]

References

[1]: Prioglio, G., Naddeo, S., Giese, U., Barbera, V., & Galimberti, M. (2023). Bio-Based Pyrrole Compounds Containing Sulfur Atoms as Coupling Agents of Carbon Black with Unsaturated Elastomers. *Nanomaterials*, *13*(20), 2761.
